# Recent Advances in Screening Methods for the Functional Investigation of Lytic Polysaccharide Monooxygenases

**DOI:** 10.3389/fchem.2021.653754

**Published:** 2021-04-12

**Authors:** Damao Wang, Yanping Li, Yuting Zheng, Yves S. Y. Hsieh

**Affiliations:** ^1^ College of Food Science, Southwest University, Chongqing, China; ^2^ Division of Glycoscience, Department of Chemistry, School of Engineering Sciences in Chemistry, Biotechnology and Health, Royal Institute of Technology (KTH), AlbaNova University Centre, Stockholm, Sweden; ^3^ School of Pharmacy, College of Pharmacy, Taipei Medical University, Taipei, Taiwan; ^4^ Genomics Research Center, Academia Sinica, Taipei, Taiwan

**Keywords:** lytic polysaccharide monooxygenase, activity, detection, analysis, methods

## Abstract

Lytic polysaccharide monooxygenase (LPMO) is a newly discovered and widely studied enzyme in recent years. These enzymes play a key role in the depolymerization of sugar-based biopolymers (including cellulose, hemicellulose, chitin and starch), and have a positive significance for biomass conversion. LPMO is a copper-dependent enzyme that can oxidize and cleave glycosidic bonds in cellulose and other polysaccharides. Their mechanism of action depends on the correct coordination of copper ions in the active site. There are still difficulties in the analysis of LPMO activity, which often requires multiple methods to be used in concert. In this review, we discussed various LPMO activity analysis methods reported so far, including mature mass spectrometry, chromatography, labeling, and indirect measurements, and summarized the advantages, disadvantages and applicability of different methods.

## Introduction

**FIGURE 1:**
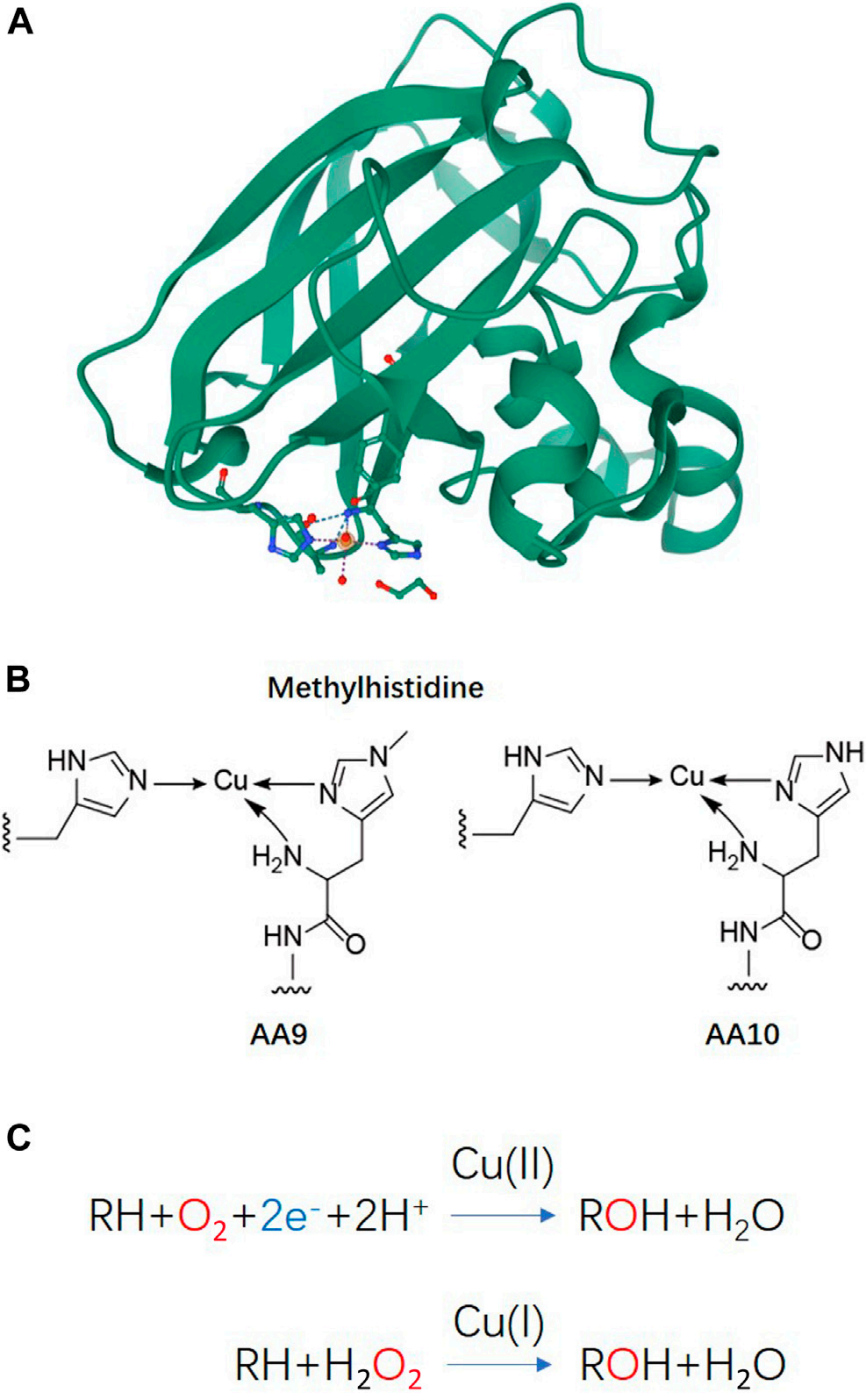
Basic structure information and reaction schemes of LPMO **(A)** Overall structure of an AA10 LPMO *Ba*AA10 from *Bacillus Amyloliquefaciens* with the active site copper shown as a sphere and active site residues shown as sticks (Gregory et al., 2016). **(B)** Schematic representations of the copper active sites observed in AA9 and AA10 structures. **(C)** LPMO reaction schemes. Cu(II)/Cu((I) indicated above the arrows refers to the copper ion in the active site and its oxidation state before initiation of the catalytic cycle.

**FIGURE 2:**
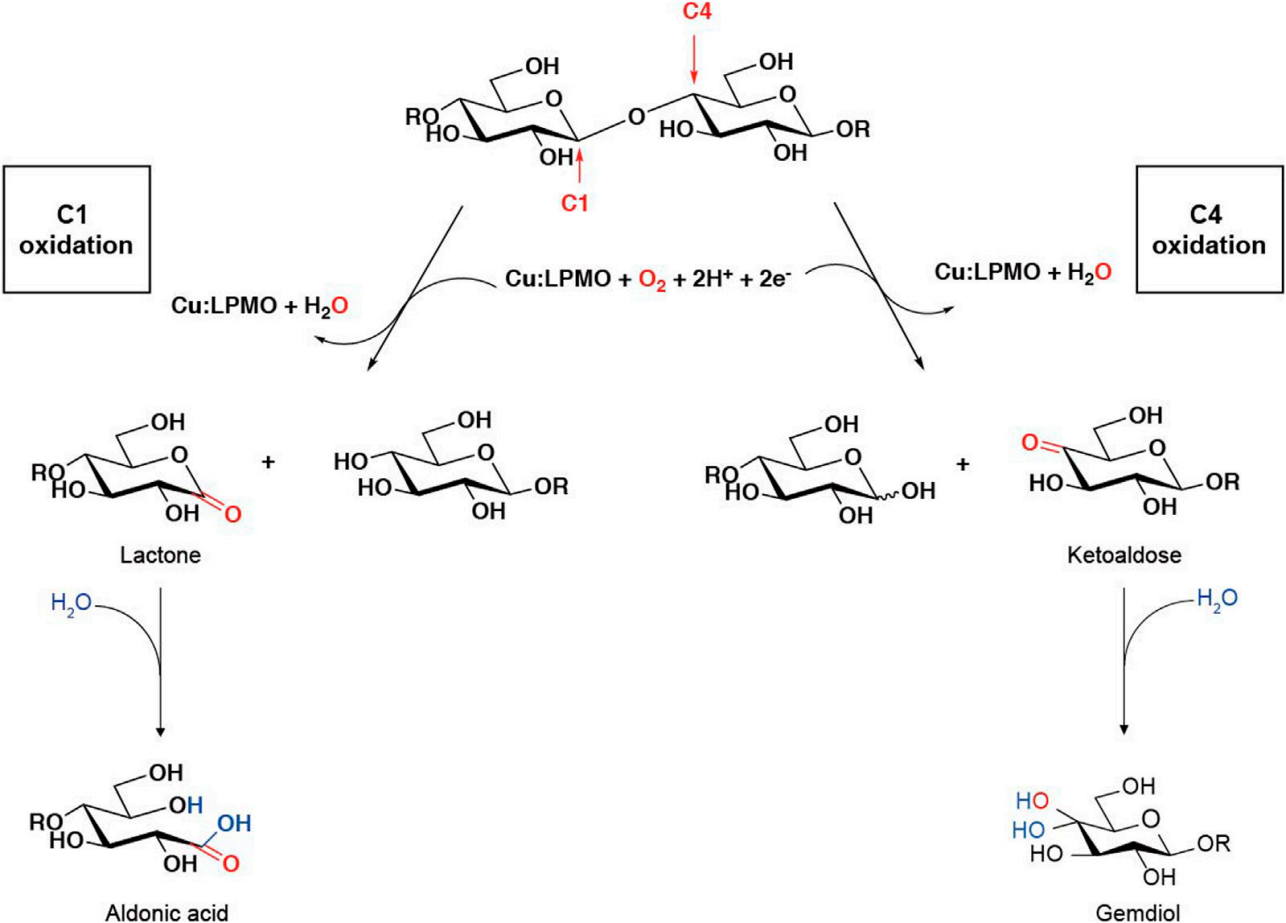
LPMO oxidation within a polysaccharide chain leading to chain cleavage. [The figure is modified on the basis of (Beeson et al., 2012) and (Hemsworth et al., 2015)].

Lytic polysaccharide monooxygenases (LPMOs), also known as auxiliary activity (AA) family enzymes, are powerful tools in the degradation of plant and marine biomasses, including cellulose (Quinlan et al., 2011; Eibinger et al., 2014; Harris et al., 2014), hemicellulose (Agger et al., 2014) chitins (Vaaje-Kolstad et al., 2010; Bissaro et al., 2020; Nakagawa et al., 2020), heteroxylans (Couturier et al., 2018; Huttner et al., 2019; Zerva et al., 2020), and starch (Vu et al., 2014b; Lo Leggio et al., 2015; Vu et al., 2019). These sustainable natural resources have been used in the production of biofuels (Hemsworth et al., 2015; Muller et al., 2015; Johansen, 2016), food-additives, packing materials (Wang et al., 2018a; Koskela et al., 2019), and added-value chemicals (Meier et al., 2018). Although degradation of recalcitrant polysaccharides using glycoside hydrolases (GHs) is a slow and complex process, the discovery of LPMOs has improved the efficiency to a great extent by boosting the activity of hydrolases towards recalcitrant cellulose and chitin (Vaaje-Kolstad et al., 2005; Merino and Cherry, 2007). The LPMO enzyme family was first discovered in 1992 (Raguz et al., 1992), but it was not until 2010 that scientist began to uncover their functional identity, which is distinct from the glycoside hydrolase family (Vaaje-Kolstad et al., 2010). The enzyme has been renamed and classified into the Auxiliary Activity family in the Carbohydrate-Active enZymes (CAZy) database (Levasseur et al., 2013; Lombard et al., 2014). To date, a total of 6 families of LPMOs have been reported, including AA9, AA10, AA11, AA13, AA14, and AA15. Most of the LPMOs have been functionally characterized to belong to family AA9–AA13, e.g., bacterial and viral LPMOs from family AA10, and fungal LPMOs from the family AA9, AA11, and AA13. Interesting, all LPMOs reported so far only act on β-1,4-linked (AA9, AA10, and AA11) or α-1,4-linked (AA13) polysaccharides. The regiospecificity is not strictly associated with families and can be substrate-dependent (Fanuel et al., 2017; Simmons et al., 2017; Bissaro et al., 2018; Chen et al., 2018). LPMOs are mono-copper associated metalloenzymes, despite having a variety of substrate specificities, they all have a catalytic mono-copper within the center of its “histidine-brace” positioned on a flat enzyme surface and an immunoglobulin-like β-sandwich fold in the core of the protein (Quinlan et al., 2011; Walton and Davies, 2016) (Figures 1A,B). This unusual structural characteristic offers the LPMOs remarkable oxidative power (Hemsworth et al., 2013; Meury et al., 2017; Ciano et al., 2018). The main effect caused by LPMOs is the oxidation of either C1 or C4 position of glycosidic linkages on the polysaccharide substrates (Figure 2) (Phillips et al., 2011; Beeson et al., 2012; Vu et al., 2014a; Isaksen et al., 2014), leading to a chain cleavage and therefore exposing more chain-ends as “access point” to the GHs, resulting in a boost of the saccharification process (Horn et al., 2012; Dimarogona et al., 2013).

In order to harness the oxidative power of LPMOs, an external electron donor is required along with the co-existence of oxygen or hydrogen peroxide and a substrate to complete the catalytic reaction (Figure 1C) (Bissaro et al., 2017; Meier et al., 2018; Müller et al., 2018). In nature, a variety of phenolic compounds produced by the degradation of lignocellulose could serve as electron donors to LPMO catalysis (Frommhagen et al., 2017; Frommhagen et al., 2018b). Cellobiose dehydrogenase (CDH) has also been reported as a natural electron donor (Phillips et al., 2011; Tan et al., 2015). Synergistic action with other enzymes, such as formaldehyde oxidoreductase (Martínez et al., 2017), polyphenol oxidase (Frommhagen et al., 2017), laccase (Brenelli et al., 2018) or the oxidoreductase family of GMC oxidoreductase (Kracher et al., 2016), can also provide a variety of potential electron donor systems for LPMO by using the released or recycled phenolic lignin breakdown products.

LPMO’s catalytic activity has been thought to be dependent on the availability of O_2_. However, recent studies revealed that LPMOs prefer H_2_O_2_ over O_2_ as a co-substrate, that the low catalytic activities observed in previous studies were most likely due to an absence of endogenous H_2_O_2_ (Bissaro et al., 2017; Caldararu et al., 2019). Specifically, kinetic investigation of bacterial and fungal LPMOs showed that the turnover numbers with H_2_O_2_ as co-substrate significantly exceeded those obtained with O_2_ by at least two orders of magnitude (Hangasky et al., 2018; Kuusk et al., 2018). It is suggested that the peroxygenase reaction may make LPMOs susceptible to oxidative damage in absence of a substrate or when H_2_O_2_ concentration is high; the lower turnover with O_2_ could instead protect the LPMO from such oxidation reactions and thus extend its operational stability for longer (Petrovic et al., 2018). These new findings that LPMO redox activity can be enhanced by H_2_O_2_ alone, and the activity is proportional to the concentration of hydrogen peroxide have a significant impact on their use in biorefineries.

Compared to glycoside hydrolase, the measurement of LPMO catalytic properties is not as straightforward. Firstly, only small proportions of soluble aldonic acid/ketoaldose oligomers are released after catalysis, whereas the majority of oxidized products remained intact with the insoluble substrate. Secondly, referring to LPMO mediated C1 and/or C4 oxidation, this may not be easily distinguishable by mass spectrometry (MS) because of the ions could be derived from a mixture of isomeric metal-adducts. Finally, the oxidative power of LPMO is directly proportional to the oxidized insoluble polysaccharide substrate, but the qualitative and quantitative analyses on insoluble substrates have always been the nodus (Eijsink et al., 2019). In recent years, a variety of methods have been developed for the investigation of LPMO activity analysis and detection (Westereng et al., 2018), including in-depth development and improvement based on traditional chromatography, mass spectrometry, and rapid detection methods suitable for many different applications. In this review, we will summarize the methodologies currently developed for the LPMO research.

## Credible Qualitative and Quantitative Analysis by MALDI-TOF-MS and HPAEC-PAD

The detection of soluble products is easier than that of the insoluble part. Moreover, there is currently no discovery of LPMO that only oxidizes on insoluble substrates without releasing soluble oligouronic acid. The most convenient, credible, fast and effective method is Matrix-assisted laser desorption/ionization time-of-flight mass spectrometry (MALDI-TOF-MS). This method is extremely sensitive and can detect trace level substances, with a good detection effect on enzymes with relatively low activity such as LPMO, but the disadvantage is also obvious, that is, it can only do qualitative analysis rather than be quantitative. On the other hand, due to its high sensitivity, other enzymes or contaminants will very likely affect the detection results, which is manifested as high background and the appearance of miscellaneous peaks, resulting in more difficult to obtain accurate mass spectra. Therefore, it is necessary to pay special attention to the purity of the reaction system, and the necessary control group also needs to be set, such as whether there is a reducing agent, copper ion or substrate. By comparing the mass spectrum results of the control groups, the activity of LPMO can be determined and identified more intuitively. Another way to set up a control group is to inactivate the enzyme, which can be simply achieved by thermal inactivation or adding a divalent metal ion chelating agent, such as EDTA (Xiao and Wedd, 2010).

Sometimes we can judge the relative amount of products by intuitive impressions, such as whether it is easy to obtain the mass spectrum, the relative abundance, etc. It is worth noting that, as shown in Figure 2, the glycosidic bond cleavage with C1 oxidation leads to the formation of one chain end, which is a lactone and a regular nonreducing end. The lactone is in equilibrium with the aldonic acid form which dominates at neutral pH. The glycosidic bond cleavage with C4 oxidation leads to the formation of a new regular reducing end, whereas the nonreducing end is a 4-ketosaccharide which is hydrated to the corresponding gemdiol form in aqueous conditions, resulting in an identical mass of certain oligos of lactone and 4-ketoaldose as well as their hydrated form. For C1 oxidation, the aldonic acid form is charged and thus tends to form double metal cation adducts, while this dication adduct peak will not appear in LPMO with only C4 oxidation activity (Frommhagen et al., 2016). It is more complicated to distinguish the double oxidation activity of C1-C4. Compared with the single C1 oxidation activity, the C1-C4 shows a higher signal to the relatively stable 4-ketone form and/or the double oxidation form of sodium adduct (Westereng et al., 2018). On the other hand, the presence of potassium ions will have a greater impact on the MALDI analysis of LPMO, which is manifested as a confusion of the molecular weights of different types of products. This issue is discussed in detail in another excellent LPMO review paper (Eijsink et al., 2019). Therefore, it is necessary to avoid mixing potassium ions in the process of LPMO reaction and preparation of test samples, which makes the analysis of enzyme activity difficult.

In 2012, a well-designed evaluation of HPLC-based analytical methods was performed 2 years right after LPMO was first reported (Westereng et al., 2013), including high-performance anion-exchange chromatography with pulsed amperometric detection (HPAEC-PAD), hydrophilic interaction chromatography (HILIC) and porous graphitized carbon liquid chromatography (PGC-LC). Each method has certain advantages, HPAEC provides quick profiling and excellent sensitivity while being able to separate relatively high DP species. PGC-LC is applicable to a variety of elution systems and can separate lactone from aldonic acid. HILIC has similar advantages as PGC-LC but has a shorter separation time and the ability to separate longer oligosaccharides than the PGC-LC. If connected to the MS module, the HILIC-MS further improves the sensitivity, with the ability to detect co-eluting peaks, which is more applicable when analyzing complex mixtures. The HPAEC-PAD is currently the most widely used method for the profiling of C1 oxidized products because, under alkaline eluent, the equilibrium tend to shift toward to the aldonic acids, C1 oxidized products are stable and remain negatively charged, so it can be separated from neutral oligomers. The C4 oxidation product is, however, unstable and decomposed during the separation process (Westereng et al., 2016), resulting in additional oxidation products. Meanwhile, under alkaline condition, C4-oxidized products were converted to native oligomers. This can be explained by the seemingly high production of native hydrolase products by C4-oxidizing LPMOs, while this phenomenon will also occur when hydrolase contaminants exist. Despite this, the C4 oxidation product will still produce characteristic and diagnostic signals in HPAEC-PAD, although the signal intensity is low due to the shift to multiple peaks and appears in the later gradient. Some LPMO will oxidize C1 and C4, indicating that they have mixed products including double oxidation products (Forsberg et al., 2014).

The purification of chromatographic samples is also an important factor affecting the analysis results. After the necessary steps such as enzyme inactivation and centrifugal filtration, solid phase extraction such as activated carbon cartridges can be used to remove impurities that interfere with chromatographic signals. LPMO reaction products can be readily identified by the retention time according to well developed and established HPAEC method. As for quantitative analysis, in-house prepared oligouronic acid standards are indispensable, but they are commercially unavailable. Oligouronic acids are prepared enzymatically, C1-oxidized cellooligosaccharide standards can be produced enzymatically using cellobiose dehydrogenase (CDH), which catalyze the oxidation of cellobiose and longer cellooligosaccharides (Bao et al., 1993; Scheiblbrandner and Ludwig, 2020) to its corresponding aldonic acid (GlcGlc1A–GlcnGlc1A) (Bennati-Granier et al., 2015; Forsberg et al., 2018), the resulting oligouronic acid products can be used standards for absolute quantification. However, the absolute quantification of the total number of oxidation sites is relatively difficult. Total hydrolysis of the substrate is needed through β-glucosidase or with other powerful commercially available hydrolase cocktails, to convert all oligouronic acids into glucose and glucuronic acid for quantification (Loose et al., 2016; Frommhagen et al., 2018a; Courtade et al., 2018) Since the C4 oxidation product is unstable and easily degraded during the analysis process, its quantitative analysis is more difficult. LPMO9C from *Neurospora crassa* can convert cellopentaose into 4-keto-cellobiose and cellotriose in equimolar ratios (Isaksen et al., 2014), so it can be quantified indirectly by cellotriose (Muller et al., 2015). NMR can also be used to analyze the activity of LPMO (Villares et al., 2017; Forsberg et al., 2019), but compared to other methods, it is more used as a product structure analysis, especially for products with overlapping molecular weights produced by the oxidation of C1, C4 and even C6, rather than comparing activities. Specifically, C1-oxidized products can be recognized by the absence of the reducing end signals (usually present at H1 α∼5.22; β∼4.66 ppm and C1 α∼94.7; β∼98.6 ppm) and more deshielded chemical shifts, while C4-oxidized products are harder to identify as they lack signals for the proton directly attached to carbon four (C4), and show minimal changes in chemical shifts for the rest of the protons, as compared to the nonoxidized monosaccharide residues (Westereng et al., 2018).

## LPMO Activity Detection Based on Insoluble Product

The above methods analyze the soluble oligouronic acid products released during the LPMO reaction but cannot analyze the effect of LPMO on the insoluble polysaccharide substrate. In the actual reaction, the ratio of soluble to insoluble products is affected by many factors, such as the setting of the reaction system, the different modes of the same substrate (e.g., Avicel and PASC), the concentration of the substrate and so on (Frommhagen et al., 2018a; Courtade et al., 2018; Loose et al., 2018). In practical applications, as an auxiliary active enzyme, the oxidative cleavage ability of insoluble polysaccharide substrates is more important, which directly determines the effect of improvement of the subsequent hydrolytic enzyme efficiency. There are also some studies using LPMO as a molecular modification tool for polysaccharides, functional groups are introduced through specific oxidation sites formed by it to obtain functional properties as in biomedicine and packaging materials. Soluble products cannot reveal the full activity of LPMO. Therefore, the comprehensive analysis of the activities of the two parts of LPMO, especially the activity analysis of insoluble substrates, is essential for evaluating the effectiveness of LPMO activity.

**FIGURE 3:**
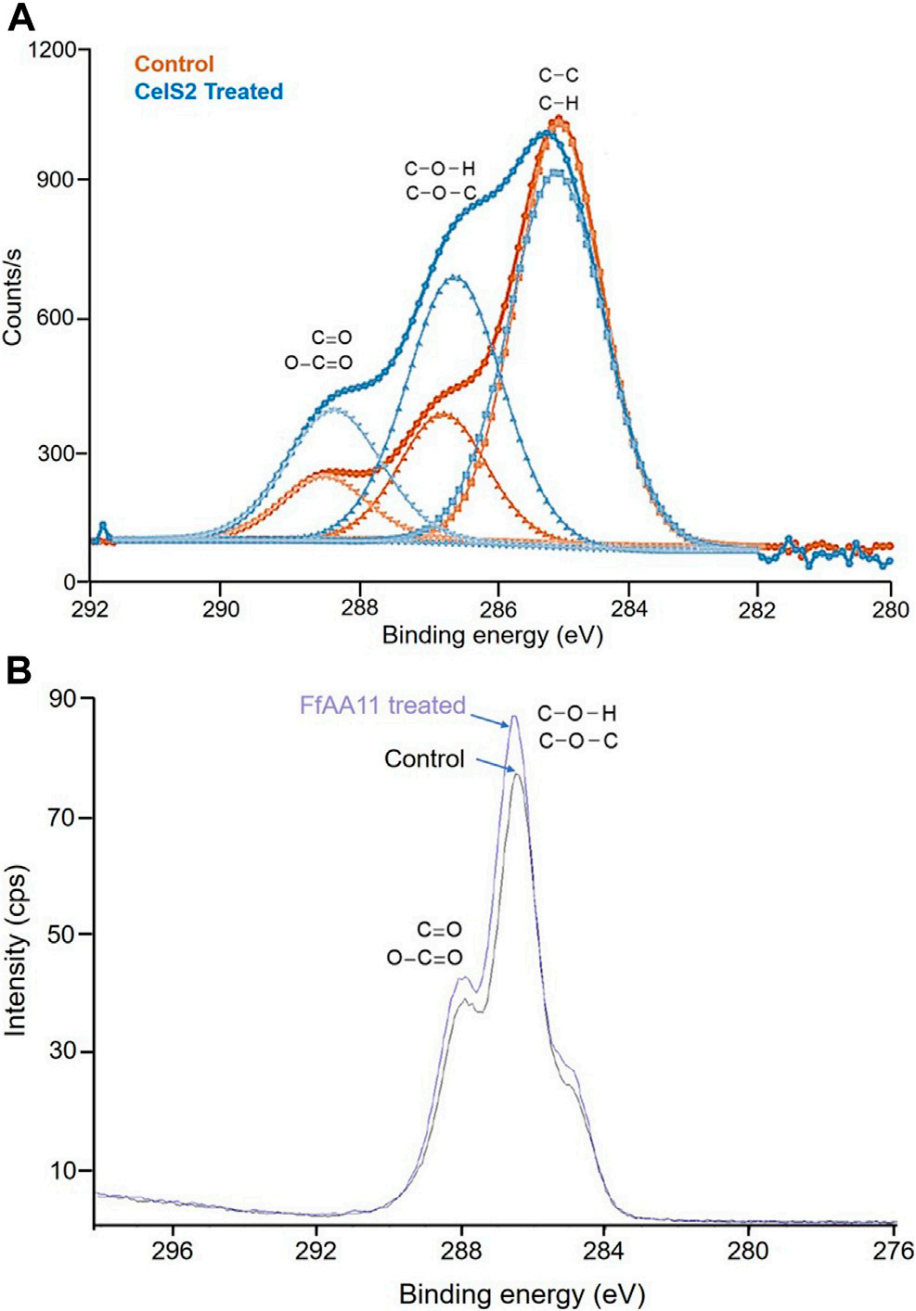
XPS analysis of LPMO treated cellulose and chitin. **(A)** C1s regions of the control and CelS2-treated sensors; circles represent experimental data; diamonds represent background; curve fitting of sub-peaks are represented by squares (C–C/C–H), triangles (C–O–H/C–O–C) and inverted triangles (C=O/O–C=O). This figure is modified from (Selig et al., 2015). **(B)** XPS comparison **(bottom)** between C 1s spectra of reference chitin (black) and LPMO-treated chitin (purple). This figure is reproduced from supporting information (Wang et al., 2018a).

X-ray photoelectron spectroscopy (XPS) can accurately measure the inner electron binding energy of atoms and their chemical shifts to provide information on molecular structure and atomic valence for chemical research. And it can provide not only general chemical information but also surface, micro-region and depth distribution information. The oxidation of polysaccharide by LPMO introduces oxygen atoms into the aldose and this molecular structural change can be detected by XPS. Michael J et al. applied XPS to verify the activity of LPMO on an insoluble substrate (Selig et al., 2015). In their study, XPS was used to verify the presence of residual oxidized cellulose on the sensor after LPMO treatment. This analysis shows that the photoelectron peak of the processed sensor has changed significantly (Figure 3A). In particular, the atomic percentage of the C=O/O–C=O bond type increased from 10% in the control to 17% in the LPMO-treated sample. This increase in C=O/O–C=O peak area supports the oxidation of LPMO and the presence of cellulose on the sensor surface. Due to the high cost of using XPS, there are fewer references available. The limitations of this method are also reported in the few papers using XPS to analyze LPMO activity. For example, Wang et al. used XPS to analyze the surface carboxylate groups of insoluble chitin (after removing enzymes and dissolved sugars). The enzymatic formation of C-1 carboxylic acid on N-acetyl glucosamine (GlcNAc) can be assessed by the increase in the abundance of the corresponding C4 carbon in the chitin treated with *Ff*AA11 (peak value is 289.1 eV). However, it was observed that the carbon spectra of the reference and enzyme-treated chitin were very similar, indicating that the carboxylate moiety on the chitin surface may not be enough to be detected by XPS analysis (Wang et al., 2018a; Figure 3B). Therefore, this method has not yet become the mainstream method for analyzing LPMO activity.

**FIGURE 4:**
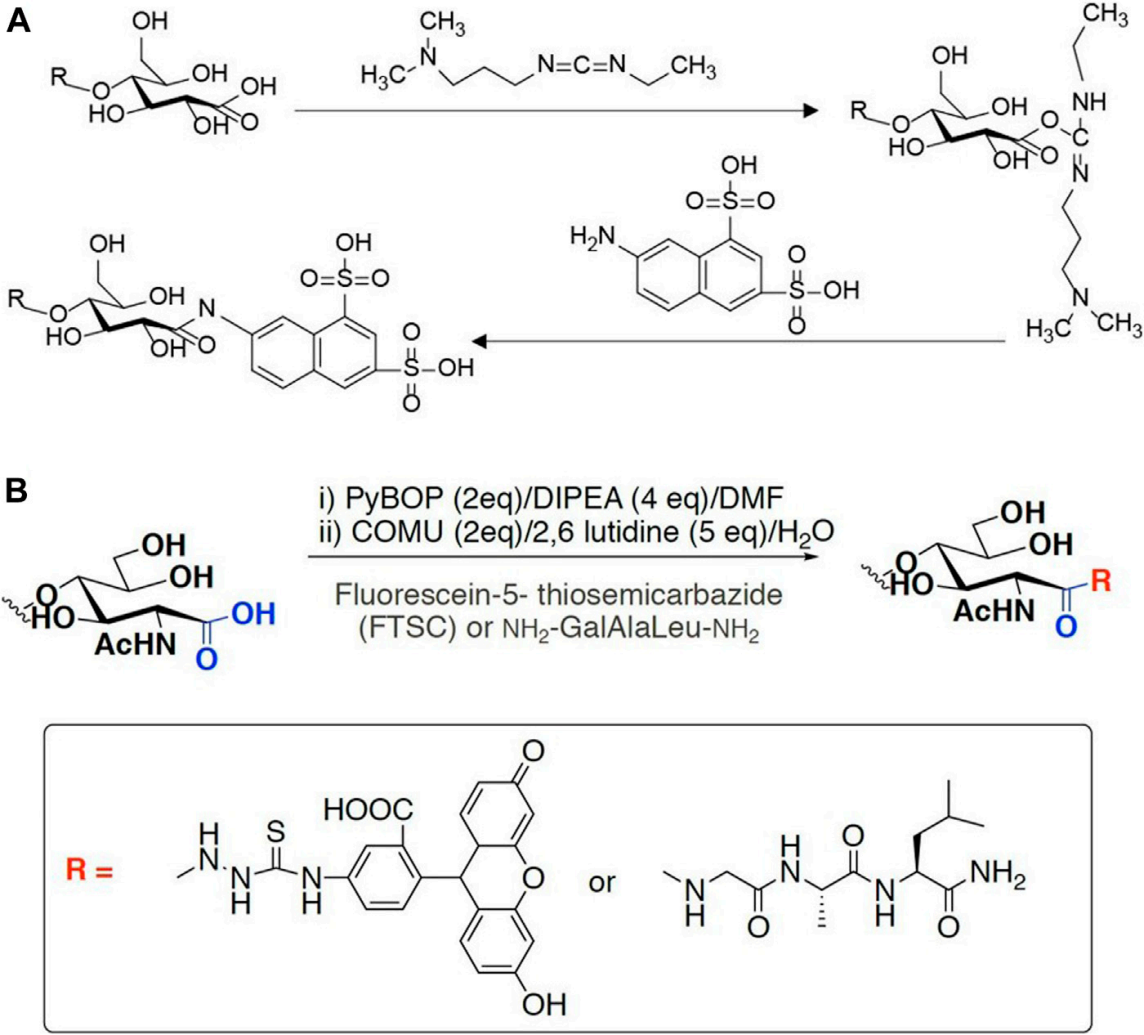
Labeling scheme of LPMO products. **(A)** fluorophore ANDA cross-link to the carboxyl group of insoluble product in the presence of EDAC. **(B)** FTSC conjugation with carboxylated chitin through amide bond formation.

Specific labeling and detection of oxidation sites are the main methods to detect the oxidation efficiency of LPMO on insoluble substrates. Commonly used labels can be fluorescent groups, radioisotopes and other easily detectable groups (Figure 4). For instance, Thu V. et al. used 1-ethyl-3-[3-(dimethylamino)propyl]carbodiimide (EDAC), which is a water-soluble carbodiimide to couple amines to the carboxyl groups introduced by LPMO. In specific, a cheap and water-soluble fluorescence dye, 7-amino-1,3-naphthalenedisulfonic acid (ANDA), was conjugated to the carboxylated cellulose for further screening by fluorescence intensity measurement (Vuong et al., 2017). The results showed that after ANDA labeling, the intensity of the fluorescence signal was positively correlated with the concentration of the cellulose substrate oxidized by LPMO. Meanwhile, in the absence of EDAC, there was no significant difference of fluorescence intensity between the LPMO treatment and the control group, indicating the feasibility and effectiveness of this method. This was also confirmed by the subsequent XPS analysis. However, cellulose incubated with EDAC alone can retain low levels of ANDA, which may be due to crosslinks between ANDA and pre-existing carboxyl groups. Using radioisotope labeling, Wang et al. invented a modified radioactive labeling approach to determine the content of carboxylic acid groups in chitins treated with a C1 LPMO (Anderson and Stone, 1985). The assay was performed by conjugating ^3^H from tritiated sodium borohydride with N-Cyclohexyl-N′-(2-morpholinoethyl) carbodiimide metho-p-toluenesulfonate, followed by measuring the radioactivity using a MicroBeta2® Microplate Counter. This labeling method has high specificity, especially reducing the influence of background value on quantitative results. But the cost is relatively high, and the accessibility and popularity of the required scintillation instruments are relatively poor, therefore it may only be suitable for conditional laboratories.

**FIGURE 5:**
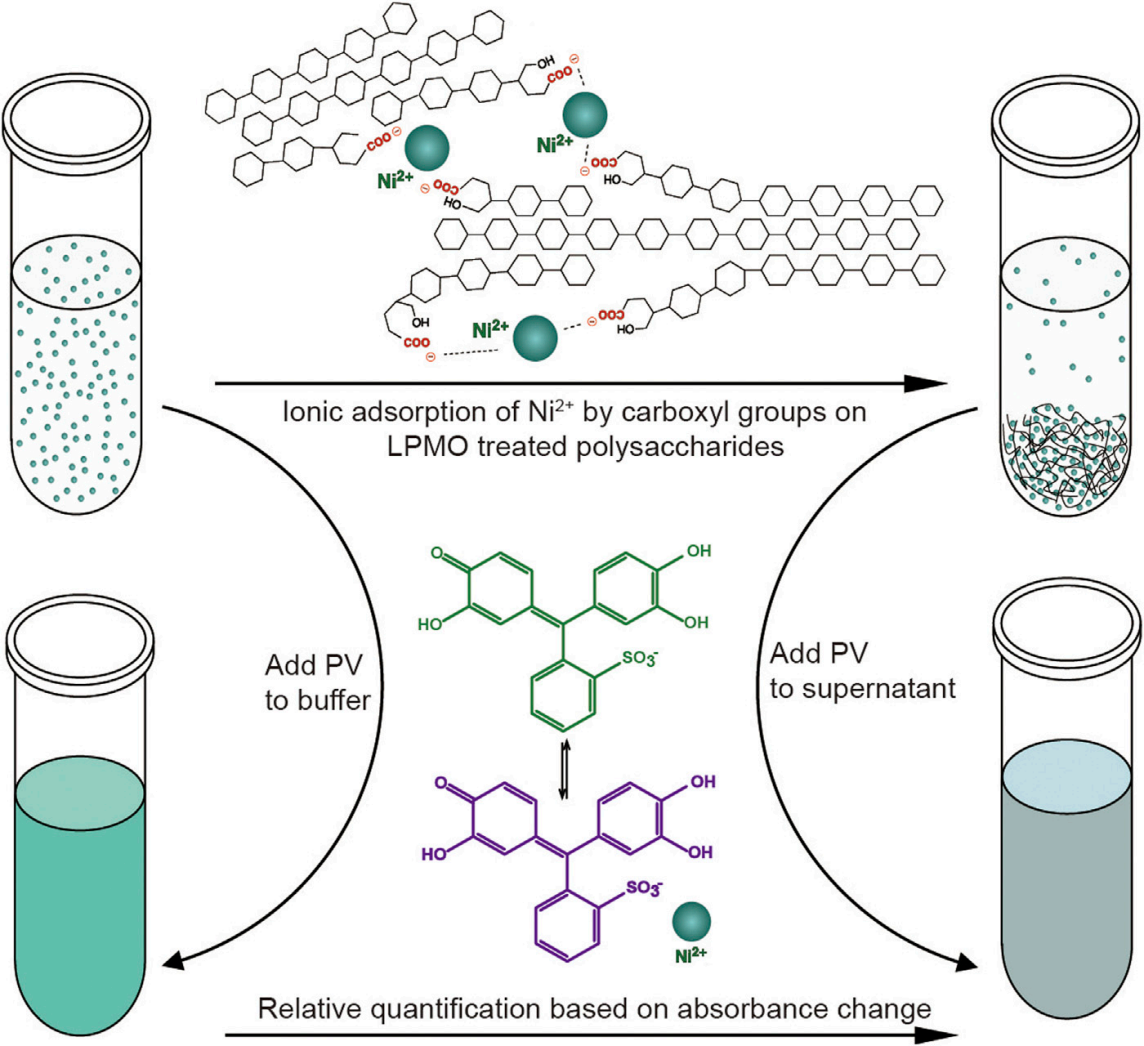
Schematic diagram of the colorimetric assay of type-1 (C1) LPMO. Figure is reproduced from (Wang et al., 2018b).

Similar to fluorescence or isotope labeling, a simpler method is based on ion adsorption, which detects the difference in the concentration of free nickel ions caused by carboxylate adsorption of nickel ions to convert the number of carboxyl groups caused by LPMO oxidation (Wang et al., 2018b). The assay is performed by incubating the insoluble reaction product (i.e., partially oxidized chitin or cellulose) with Ni^2+^, which binds to the aldonic acid groups, and quantify the remaining Ni^2+^ in the solution using a complexometric indicator pyrocatechol violet through spectrophotometric analysis. This method is very convenient and easy to use. Compared with other labeling methods, it does not require complicated chemical pretreatments (Figure 5). It is particularly suitable for high-throughput screening and rapid comparison of C1 LPMO activity. This easily adaptable and scalable assay is suitable for different types of polysaccharide substrates such as cellulose, chitin, xylan and xyloglucans, and it is even useful for soluble oligosaccharides since they can be fully precipitated by ethanol. However, this method also has obvious limitations. For example, the Ni^2+^-carboxylate complex does not follow a 1:2 ratio as the spacing between the carboxylate moieties introduced by LPMO cannot be precisely regulated, and the carboxymethyl cellulose used as a standard may be inappropriate since it may differ from the substrate in micro-scale morphology. Although this method has limitations in absolute quantification, it is still a very simple and universal method for rapid relative quantification and activity screening. In addition to labeling the products, researchers also use specially processed polysaccharide substrates for enzymatic reactions to more accurately monitor the activity and reaction mode of LPMO. Frandsen et al. developed an assay that used derivatized cellotetraose to measure LPMO activity and showed a FRET quenching effect, which was relieved after the oligomeric substrate was cleaved (Frandsen et al., 2016). This method is sensitive and accurate, but it is overly costly and is only suitable for the activity identification of small amounts of oligosaccharide substrates. In addition, scientists have engineered chromogenic substrates (Vidal-Melgosa et al., 2015), such as chromogenic polysaccharide hydrogel which can qualitatively identify the activity of LPMO in efficient manner (Kracun et al., 2015), but this type of methods is more suitable for high-throughput substrate-specificity screening. The above methods for analyzing LPMO activity by indirect analysis are simple and easy to use, however, they can only be used as auxiliary analysis methods since they cannot directly obtain conclusive product information, especially for biomass conversion which is the main application of LPMO. In general, newly discovered LPMOs need to be determined by conclusive product analysis, such as MS or HPAEC, and then rapid activity comparisons can be performed through the above indirect analysis methods, then again, since these methods are easily affected by many factors, indirect analysis alone is not conclusive.

## Indirect Detection Methods Not Based on Measuring Reaction Products

There are methods that do not directly target the LPMO reaction product, but indirectly measure the changes of other parameters brought about by the LPMO reaction to detect the activity of LPMO. Electron paramagnetic resonance (EPR) is a magnetic resonance spectrometry derived from the magnetic moment of unpaired electrons. It can be used to qualitatively and quantitatively detect the unpaired electrons contained in the atoms or molecules of substances and the structural characteristics of the surrounding environment, including the mechanism of enzyme action on insoluble and stubborn biomass (Liao et al., 2014). Cianl et al. developed a simple experimental semi-directional EPR method to evaluate the binding of LPMO to crystalline cellulose (Ciano et al., 2020). The enzyme showed obvious angle-dependent changes in its EPR spectrum, indicating the partial orientation of the protein on the substrate. The subsequent angle-related changes observed in the EPR spectrum can be related to the orientation of the main direction of the *g* matrix with respect to the magnetic field of the spectrometer, and therefore to the binding of enzymes to cellulose fibers. This methodology allows us to expand our current knowledge of the effects of LPMO on refractory substrates and can be used as a general method to assess the binding of LPMO to substrates, and it can also indirectly confirm its activity.

Kojima et al. tried to analyze the activity of LPMO by observing the dynamic viscosity changes of the substrate caused by LPMO, and successfully observed the viscosity decrease when using xyloglucan, glucomannan, arabinoxylan, and carboxymethyl cellulose as a substrate (Kojima et al., 2016). The limitation of this simple and universal method is that it can only measure soluble polysaccharides but not insoluble polysaccharides, especially the two most important biomass of cellulose and chitin.

**FIGURE 6:**
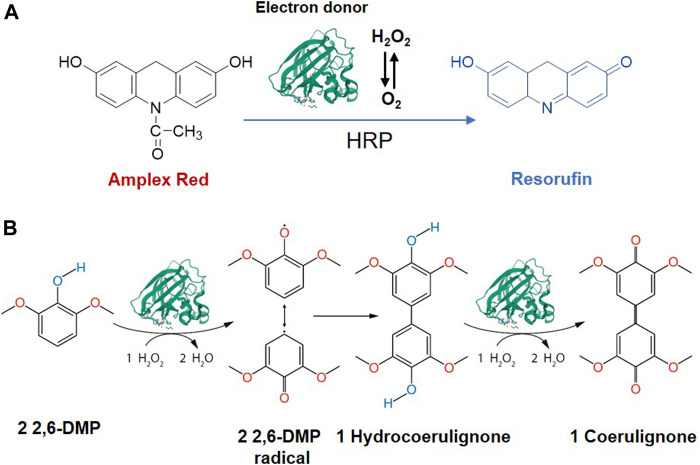
LPMO assays based on its peroxygenase activity. **(A)** Ascorbate or CDH reduce the Cu(II) in the LPMO, oxygen was activated by electron reduction. Released H_2_O_2_ is detected by the HRP coupled conversion of Amplex Red to resurofin. **(B)** Oxidation of 2,6-DMP to the corresponding phenoxy radical at the expense of H_2_O_2_ and LPMO. Cu(II) is reduced by 2,6-DMP which generates the 2,6-DMP radical. The radicals dimerize rapidly to hydrocoerulignone, which is again quickly converted to coerulignone by LPMO. The figure is reproduced and modified from (Kittl et al., 2012) and (Breslmayr et al., 2018).

LPMOs is reduced in the presence of O_2_, generating H_2_O_2_ which is detectable in solution. Therefore, assays based on the peroxidase activity of LPMO were developed (Figure 6). Ascorbate or cellobiose dehydrogenase (CDH) reduce the type-2 copper in the LPMO, which activates molecular oxygen by a one-electron reduction. The released H_2_O_2_ is detected by the HRP coupled conversion of Amplex Red to resurofin which can be detected spectrophotometrically (ε_571_ = 58,000 M^−1^ cm^−1^) or fluorimetrically (Ex = 569 nm/Em = 585 nm). However, this assay requires a high LPMO concentration (20–574 mg L^−1^) to make reliable measurements, which is not acceptable for LPMOs since they are not generally expressed in such high abundance. Besides, the uncoupling reaction is slow and the sensitivity is low, meanwhile, it is also susceptible to the influence of metal ions present in the fermentation medium, which requires re-buffering or diafiltration to eliminate this effect. Therefore, the Amplex Red-based assay is only suitable for rapid activity measurement to quantify LPMO activity during protein purification. It is not suitable for screening LPMO activity in culture supernatant, characterizing mutation changes, or measuring physiological responses. In 2018, an improved assay based on the peroxidase activity and 2,6-dimethoxyphenol (2,6-DMP) oxidation was developed. LPMO catalyzes the oxidation of 2,6-DMP to the corresponding phenoxy radicals at the expense of H_2_O_2_. The active-site Cu(II) is reduced by 2,6-DMP which generates the 2,6-DMP radical. Two formed 2,6-DMP radicals dimerize rapidly to hydrocoerulignone, which again is quickly converted to coerulignone by LPMO. This activity can be accurately measured for rapid screening of enzyme activity as well as to study the binding constants or thermal stability. In 2019, a more developed assay following the colorimetric oxidation of hydrocoerulignone instead of 2,6-DMP to coerulignone was invented, which reduces the inhibition effect by different buffer species and performs faster (Breslmayr et al., 2019). Since oxygen is another substrate in the LPMO catalysis process, the reaction process and activity of LPMO can be evaluated by measuring the change in dissolved oxygen content. Cannella et al. found that oxygen is consumed by LPMO incubated with cellulose in a light-induced system, by comparing the oxygen consumption rate of different LPMOs, a quick comparison of enzyme activities can be achieved (Cannella et al., 2016).

### Detection of LPMO Activity by Visualizing the Reaction Products

In addition to the above direct or indirect analysis methods, observation of the microscopic morphological changes of the substrate occurred during the LPMO catalytic process through imaging methods can also be used as means of qualitative analysis. It is showed that after LPMO treatment and fluorescent staining, the cellulose substrate exhibited transmission and fluorescence under a laser confocal microscope, while the control group did not exhibit this phenomenon (Eibinger et al., 2014). This method is suitable for the qualitative analysis of LPMO activity. At the same time, observing the position of the fluorescent signal on the substrate can help to determine the selectivity of LPMO. Similarly, atomic force microscopy (AFM) can also be used to observe subtle changes in the substrates caused by LPMO oxidation. Through real-time image analysis, researchers found that single LPMO molecules move intermittently and randomly along the cellulose ribbon-like microfibers, accompanied by the release of a small amount of oxidized sugars and the splitting of large cellulose ribbons into fibrils. Although LPMO only promoted the degradation of the cellulose surface structure to a small extent, the enzymatic activity was detected on the crystalline areas of the substrate, and these areas were removed at a slow rate while the surrounding amorphous material areas were not affected. The degradation of the fibrils is mainly carried out by thinning from the sidewalls, but also by cracking in the middle to form smaller and shorter fragments (Song et al., 2018). The discrete crystal surface structure is thus dissolved, resulting in the formation of oxidized oligosaccharides that can be detected in the solution (e.g., D-gluconic acid). Although this kind of microscopic morphological observation method is not quantitative, it is very helpful as qualitative analysis, especially for the analysis and judgment of the enzyme reaction process and mode of action. There are also some methods to visualize the soluble oligouronic acid produced by LPMO oxidation. For example, polysaccharide analysis using carbohydrate gel electrophoresis (PACE) (Goubet et al., 2002; Frandsen et al., 2016) or thin-layer chromatography (TLC) (Frandsen et al., 2016; Chen et al., 2018; Valls et al., 2019), these methods can only detect substrate degradation by the enzymes but cannot distinguish between oligosaccharides and aldonic acids. In conclude, based on the techniques that we discussed above, we have summarized their advantages, disadvantages and applicability to guide readers to choose the appropriate method according to their own needs and actual conditions (Table 1).

**TABLE 1:** Summarized advantages and disadvantages of the methodologies.

Features methods	Qualification or quantification	Targets (soluble/insoluble)	Stability	Accuracy	Ability of activity comparison	Quickness/Convenience
MALDI-TOF-MS	Qualification	Soluble	High	High	Weak	High
HPAEC-PAD	Both	Soluble	High	High	High	Weak
XPS	Both	Insoluble	High	High	High	Weak
Fluorescent labelling	Quantification^a^	Insoluble	Medium	Medium	Medium	High
Isotope labelling	Quantification^a^	Insoluble	Medium	High	High	Weak
Nickel ions absorption	Quantification^a^	Insoluble	Weak	Medium	High	High
FRET quenching	Quantification^a^	Soluble	Medium	Medium	High	Weak
Viscosity changes	Quantification^a^	N.a.^b^	Medium	Medium	Medium	High
Peroxidase activity	Quantification^a^	N.a.^b^	Medium	Medium	High	High
Confocal/AFM	Qualification	Insoluble	High	Weak	Weak	Medium

^a^ Here means a credible method which detect the LPMO’s product is necessary prior to this assay.

^b^ Not available. This assay not target on detection of the soluble or insoluble oxidation products.

**FIGURE 7:**
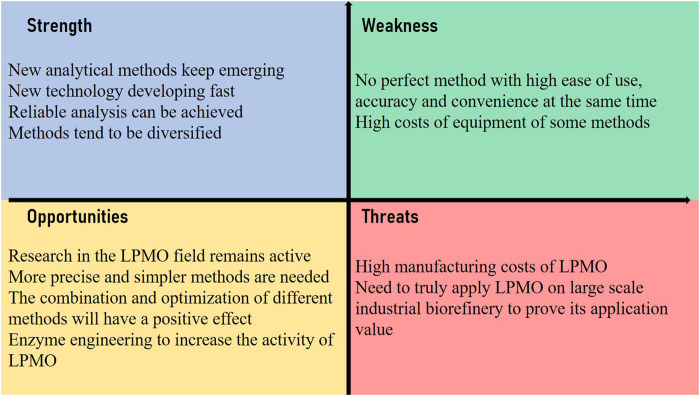
SWOT analysis of the methodology development of LPMO’s activity detection and comparison.

The conversion of biomass into fuels and other chemicals is the key to ensure a sustainable low-carbon economy in the future. The discovery of LPMO has greatly promoted the revolution of enzymatic biomass conversion and its industrial applications. The development of LPMO activity detection methods is also full of opportunities and challenges (Figure 7). Now LPMO has become a key component of today’s most advanced cellulase mixture, and the new AA family of LPMO has been discovered in various biological environments. At present, more and more scholars are engaging in LPMO research. As our understanding of the redox catalysis and deactivation of LPMOs continues to deepen, new scientific questions are raised, such as whether their overall structures are characteristically similar? What is the relationship between their substrate specificity and structure? How does N-terminal histidine methylation affect LPMO activity? The various complex reactions that occur when using mixed substrates and their relationships, etc. These issues are unresolved, continuous research efforts and advancement in methodologies are required to achieve major breakthroughs in the application and theoretical level of LPMO studies.
